# The placebo effect shortens movement time in goal-directed movements

**DOI:** 10.1038/s41598-022-23489-y

**Published:** 2022-11-15

**Authors:** Mirta Fiorio, Bernardo Villa-Sánchez, Filippo Rossignati, Mehran Emadi Andani

**Affiliations:** 1 Department of Neurosciences, Biomedicine and Movement Sciences, University of Verona, 37131 Verona, Italy; 2 Center for Mind/Brain Sciences (CIMeC), University of Trento, 38068 Rovereto, Italy

**Keywords:** Cognitive neuroscience, Motivation, Motor control, Human behaviour

## Abstract

The placebo effect is a powerful psychobiological phenomenon whereby a positive outcome follows the administration of an inert treatment thought to be effective. Growing evidence shows that the placebo effect extends beyond the healing context, affecting also motor performance. Here we explored the placebo effect on the control of goal-directed movement, a fundamental function in many daily activities. Twenty-four healthy volunteers performed upper-limb movements toward a target at different indexes of difficulty in two conditions: in the placebo condition, an electrical device (inert) was applied to the right forearm together with verbal information about its positive effects in improving movement precision; in the control condition, the same device was applied along with verbal information about its neutral effects on performance. Interestingly, we found shorter movement time in the placebo compared to the control condition. Moreover, subjective perception of fatigability was reduced in the placebo compared to the control condition. These findings indicate that the placebo effect can improve the execution of goal-directed movements, thus adding new evidence to the placebo effect in the motor domain. This study could inspire future applications to improve upper-limb movements or in clinical settings for patients with motor deficits.

## Introduction

Many daily activities, like picking up a cup of coffee or reaching a bottle of water, require an optimal balance between movement precision and speed. The speed-accuracy tradeoff for goal-directed actions has been described by Paul Fitts in 1954^1^. More precisely, Fitts demonstrated that the time needed to execute a movement with the upper limb from a starting point to a final one depends on the distance and the size of the target to be reached. The mathematical model used to describe this relationship is known as Fitts’ law: *MT* = *a* + *b log2(2A/W)*, where *MT* denotes the movement time, *a* and *b* are empirical constants, *A* is the distance between the starting point and the target and *W* corresponds to target width^1^. The ratio between the amplitude and the width (2A/W) represents the index of difficulty of the movement. Accordingly, the index of difficulty is high when the target is far and small, whereas it is low when the target is near and big. The index of difficulty has an impact on the movement time: a higher index of difficulty is typically associated with a longer movement time, while a lower index of difficulty is associated with a shorter movement time.

Over several years, Fitts’ law has been applied in many studies to explain the speed-accuracy trade-off observed in goal-directed movements and to clarify the principles that guide movement planning and execution^2–6^. Moreover, Fitts’ pointing task revealed to be useful to investigate the effects of different factors, like age^7,8^, fatigue^9^, and social context^10^ on movement execution. According to optimal control models, Fitts’ law emerges as a consequence of an optimal strategy to successfully complete a task in the presence of signal-dependent noise^9,11^. To cope with noise, motor control benefits from anticipatory processes in the central nervous system, which are based on internal models of the future outcome of the action^12^. Predictive models of brain functioning^13^ posit that internal predictions about the outcome of an action can influence the way in which the action will be performed. Hence, ‘expectation’—defined here as the anticipation of a certain motor outcome—has a strong influence on action execution.

A growing body of research supports that positive expectations induced through placebo procedures result in better motor control for several motor functions—i.e., force, speed, balance, general motor learning, and resistance to fatigue—in athletes, non-athletes, and patients with motor deficits^14–26^. These effects have been related to changes in brain areas involved in motor planning and execution, like the primary motor cortex, the supplementary motor area, the dorsolateral prefrontal cortex, and subcortical structures^14,16,19,20,23,27^. In particular, it was suggested that positive expectations induced through a placebo procedure can increase the voluntary drive to the motor cortex and, consequently, enhance corticospinal excitability^19^. By leveraging Fitts’ law, we aimed at investigating whether top-down processes, like those exerted by positive expectations, can improve precision control of upper limb movements. The assumption is that if the placebo effect modulates the anticipatory processes of goal-directed movements, then the expectation of better motor control induced through the placebo procedure would result in shorter movement time at a Fitts’ law task.

For our purpose, we devised a reaching movement task toward a target and measured movement time for different indexes of difficulty. Expectations were manipulated with a placebo procedure in which an electrical device (actually inert) was applied to the participants’ right forearm together with different verbal information about its effects. By adopting a within-subject design, the very same participant performed the task once with verbal information about the positive effects of the device in improving movement precision (placebo condition) and once with verbal information about the neutral effects of the device on performance (control condition). We predicted shorter movement times in the placebo condition compared to the control condition for different indexes of difficulty. This investigation can have the twofold impact of expanding our knowledge on the pervasive nature of the placebo effect on a motor function not yet investigated in the realm of placebo (i.e., goal-directed movement) and of opening the way for new cognitive strategies based on positive expectation to improve goal-directed movements.

## Methods

### Participants

The computation of the sample size analysis was performed a priori using the software G-Power 3.1^28^. Given that there were no published data regarding the modulation of goal-directed movement with the upper limb through a placebo effect, we assumed an anticipated effect size (f) of 0.25, which is considered as “medium”^29^. Assuming this effect size, the type I error (p-value) of 0.05 to claim statistical significance, and the power of 0.9 (to be conservative in avoiding a type ΙΙ error—i.e., high probability to detect a difference between conditions, if it truly exists), the required sample size was 24. Twenty-four healthy individuals from the students’ population of the University of Verona volunteered for the study (14 women, 20 right-handed, mean age ± SD: 19.7 ± 1.0 years, education: 13.6 ± 1.0 years). All participants received information about the task and the procedure and gave written informed consent prior to the experiment. The placebo nature of the treatment was revealed at the end of the whole experimental procedure. The research project was approved by the committee for approval of research in humans (CARP) of the University of Verona and was conducted in accordance with the Declaration of Helsinki.

### Reaching task

**Figure 1:**
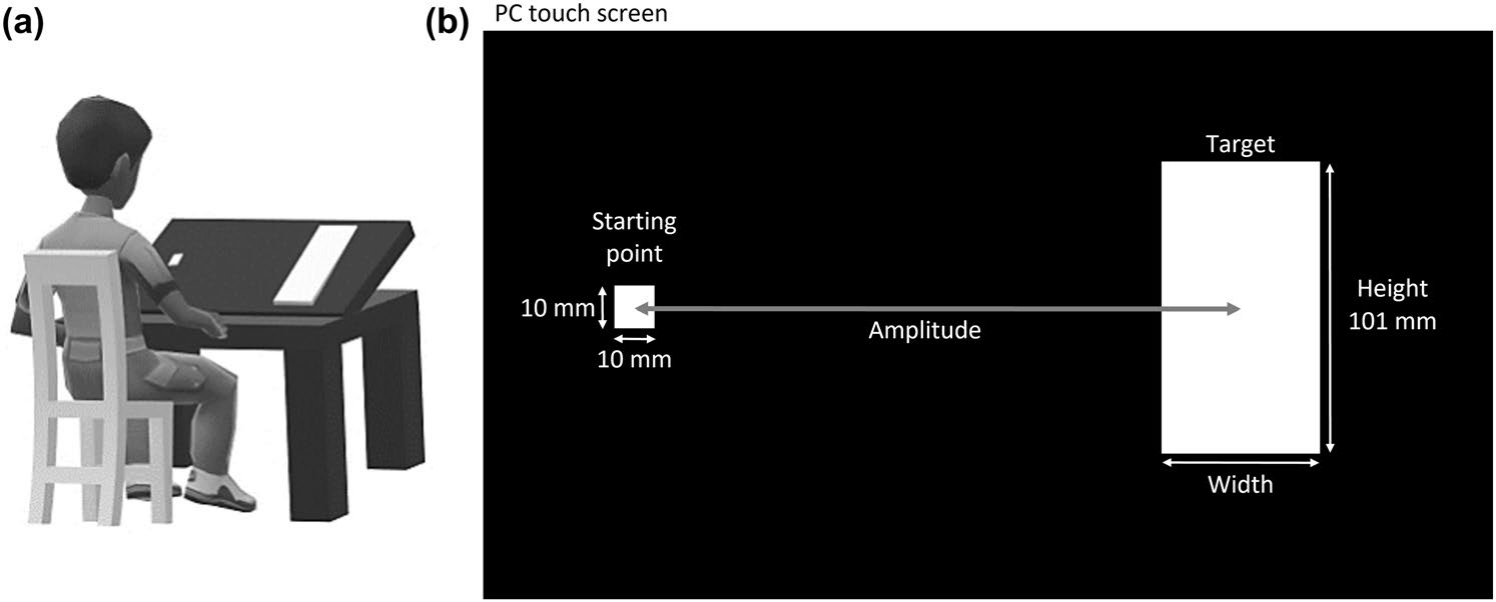
Experimental setup. (**a**) The participant’s and the PC touch screen’s positions (created by M.E.A. with Microsoft Paint 3D, URL: https://apps.microsoft.com). (**b**) Schematic representation of the reaching task from the starting point on the left to the target on the right of the PC touch screen (created by M.E.A. and M.F. with Microsoft Power Point, version 2016, URL: https://apps.microsoft.com). The combination of target widths and amplitudes defined different indexes of difficulty.

The experiment was conducted in a quiet (33 dB) and dark room (6 lx). Participants were seated at a table on a comfortable chair. A PC touch screen (ASUS Full HD 1080; model: Eee top ET2203T; PC touch screen temporal resolution: 10 kHz; PC touch screen spatial resolution: 0.25 mm; Screen dimension: 48 × 27 cm; Screen resolution: 1280 × 1024 pixels) was placed on the table with the surface pitched at 30° from the table toward the participant’s face and with the center of the screen aligned to the participant’s body midline (like^30^) (Fig. 1a).

At the beginning of each trial, participants were required to place a pen (which was detected by the touch screen) on a starting point (white square, 10 × 10 mm) appearing on the left side of the PC screen (Fig. 1b). An auditory cue (of 3 kHz and 100 ms duration^3^) signaled the beginning of the trial and was delivered concurrently with the onset of a target (white rectangle) appearing on the right side of the screen (Fig. 1b). The target could appear within a time window ranging from 1 to 2 s after participants had maintained the pen stable at the starting point. Participants were asked to move their right arm rightward as fast and accurately as possible to reach the target with the pen. At the end of each trial, the target disappeared, and participants had to position the pen again at the starting point for the next trial. The movement onset was defined as the time point in which the pen velocity reached 30 mm/s for at least 20 ms and the movement end was defined as the time point in which the pen velocity dropped below 30 mm/s for at least 20 ms^30^. The time between the movement onset and the movement end was defined as movement time (MT) and was considered as the main outcome of performance. Only MT for correct trials was entered in the analysis. Trials in which the pen passed beyond the target area to the right side, or in which the pen did not enter the target area were considered as errors^30^ and were removed from the computation of the MT. Whenever an error occurred, the trial was repeated. The percentage of error was derived from the Eq. (1).

1 $$Percentage \; of \;  Error=\frac{{N}_{Errors} }{{N}_{Total trials} } \times 100 \%$$

The target height was set at 101 mm in all trials, whereas the target width (W) and the target distance from the starting point (defined as target amplitude, A) were modified in order to obtain different indexes of difficulty (IDs) according to Fitts’ law (ID = *log*_*2*_*(2A/W)*). In particular, we defined seven IDs from a minimum of 2.5 to a maximum of 5.5, in steps of 0.5 (IDs, 2.5, 3, 3.5, 4, 4.5, 5, 5.5). The two extremes (ID 2.5 and 5.5) were defined based on the notion that the A/W range that can be studied in the laboratory is 3 ≲ A/W ≲ 33–50 (equivalent to ID range of 2.5 ≲ ID ≲ 6–6.5)^31,32^. This is due to the floor effect of MT (movement time saturation) for A/W ≲ 3 (corresponding to ID ≲ 2.5), and to the ceiling effect of error rate (movement accuracy saturation) for A/W ≳ 33–50 (corresponding to ID ≳ 6–6.5). Hence, in this study, to prevent saturation of movement time and accuracy we considered an ID range between 2.5 and 5.5. The 0.5 steps between IDs allowed us to detect small changes in performance by slightly changing the ID. To define the A and W suitable to obtain the desired IDs, we first considered a maximum A of 380 mm. This value was due to the fact that our touch screen had a length of 480 mm, and considering 50 mm margins on the left and right of the screen, the maximum A possible in our task was 380 mm. Based on this A, we defined the W needed to obtain ID 5.5 (W = 16.8 mm), 5 (W = 23.8 mm), 4.5 (W = 33.6 mm), and 4 (W = 47.5 mm). To obtained easier IDs, instead of further increasing the target width, we decreased the target amplitude. This allowed us to have more combinations of A and W, thus preventing habituation to the task. To this end, we considered W of 47.5 mm to define the A corresponding to ID 3.5 (A = 268.7 mm), 3 (A = 190 mm), and 2.5 (A = 134.4 mm). To summarize, 4 target amplitudes (A = 134.4, 190, 268.7, 380 mm) and 4 target widths (W = 16.8, 23.8, 33.6, 47.5 mm) resulted in 16 different combinations and 7 indexes of difficulty (2.5, 3, 3.5, 4, 4.5, 5, 5.5) according to Fitts’ law (Table 1). Of note, the values of A and W in our study are very close to those of a previous study in which a similar task was used^30^.

**Table 1:**
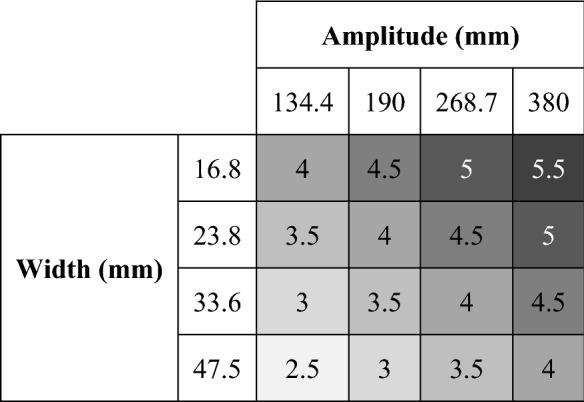
Indexes of difficulty. The combinations of 4 target widths and 4 target amplitudes resulted in 7 different indexes of difficulty (from ID = 2.5 to ID = 5.5, as reported in the cells).

A customized Matlab toolbox (MathWorks Inc.) was used to control the presentation of the different target widths and amplitudes and to continuously record the movement trajectory of the pen on the touch screen (sampled at 10 kHz). The data were saved after being lowpass filtered with a 12 Hz zero-phase second-order Butterworth digital filter. Thanks to the zero-phase filtering method, the filtered data do not shift in time when compared to the raw data, thus ruling out any potential bias in the detection of movement onset and allowing a precise computation of movement time.

### Procedure

**Figure 2:**
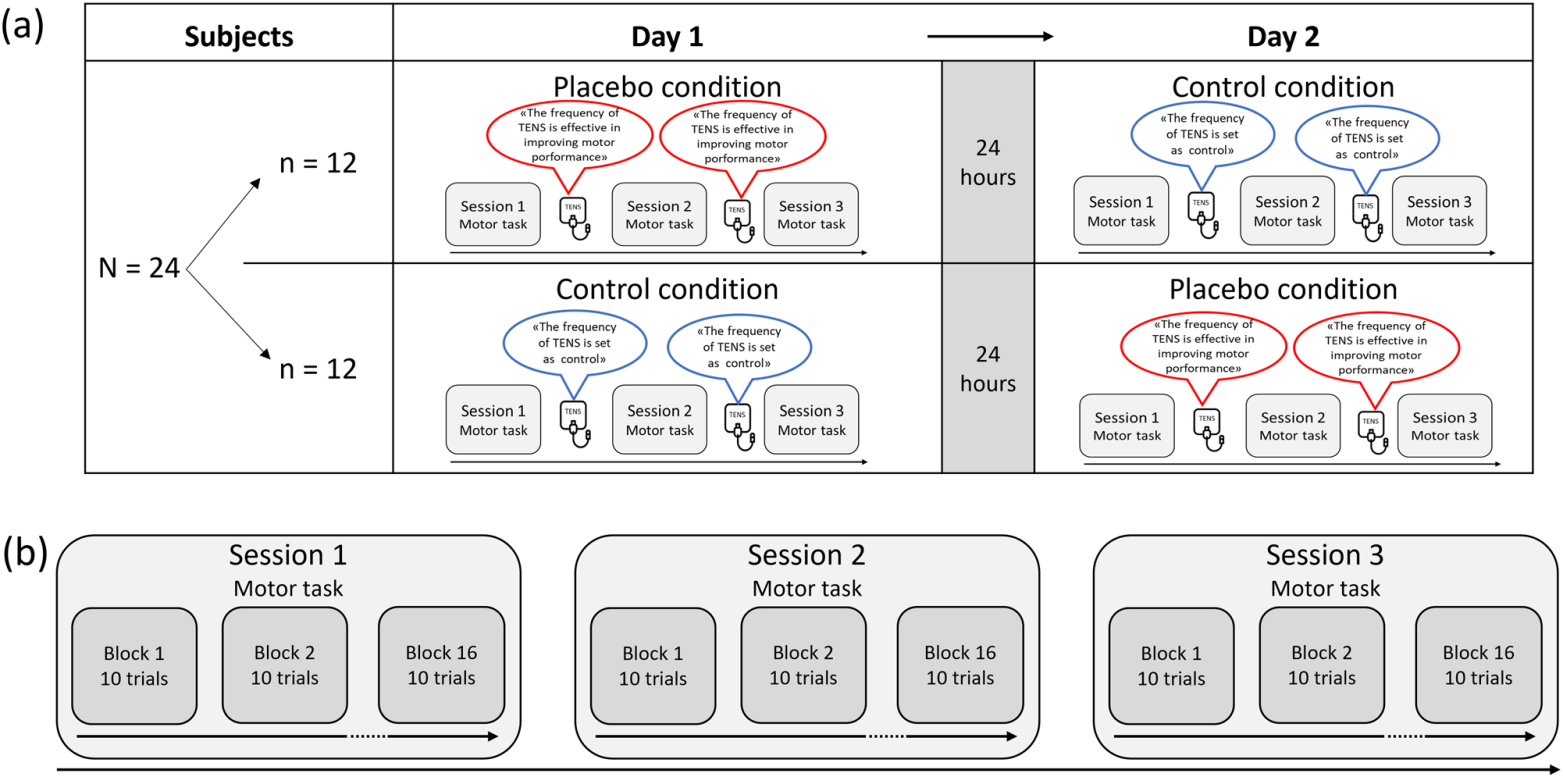
Schematic representation of the experimental design and protocol. (**a**) One group of 24 participants was tested with a within-subject, crossover and counterbalanced design. Twelve subjects underwent the placebo condition on day 1 and the control condition on day 2, while other 12 subjects had the reversed order of conditions. In each condition, the motor task was performed in three sessions: Session 1, session 2, and session 3. Before sessions 2 and 3, TENS was applied with different verbal information for the placebo and control conditions. (**b**) Each session was made of 16 blocks of 10 trials each. Each block corresponded to a combination of target amplitude and width (see Table 1). The order of the blocks was pseudo-randomized. (**b**) Schematic representation of the procedure.

We devised a within-subjects, crossover design, in which all participants underwent two conditions (placebo and control) separated by 24 h with counterbalanced order across participants (Fig. 2a). Before starting the real experiment, 10 practice trials with randomly selected target amplitudes and widths allowed participants to familiarize with the task. Each condition (placebo and control) consisted of three sessions (sessions 1, 2, and 3). Session 1 allowed us to measure performance before any intervention (baseline). In session 2, an electrical device (i.e., Transcutaneous Electrical Nerve Stimulation, TENS) was applied over the right arm before task execution, with specific verbal information depending on the condition (placebo or control, see below) (Fig. 2a). In previous placebo studies, the same device was applied for 3 to 5 min^19,24,26,27,33,34^. Hence, to remain within the same time window, TENS was applied here for 4 min. In session 3, the TENS device was applied again with the same information (placebo or control), before task execution. The second TENS application allowed us to investigate any potential strengthening of the placebo effect.

Each session was made of 16 blocks (one for each combination of target amplitude and width) of 10 trials each, for a total of 160 trials per session (Fig. 2b). A break of 10 s was inserted between blocks. The order of the blocks was pseudo-randomized so that blocks with easy IDs (2.5, 3, 3.5, and 4) were inserted between blocks with difficult IDs (4, 4.5, 5, and 5.5), with ID 4 (which was present in four combinations) distributed randomly across easy and difficult blocks. This approach allowed us to distribute any potential effect of fatigue throughout the task^30^.

### Verbal information and placebo manipulation

The covert story was that the aim of the study was to investigate the effects of an electrical device, Transcutaneous Electrical Nerve Stimulation (TENS), in improving motor performance and that to accomplish this aim we had to apply the device in two different days with different frequencies of stimulation. Unbeknown to the participants, however, TENS was applied in both days with the same frequency of stimulation (i.e., 10 Hz), which is not able to induce active modification of performance per se, as shown in previous studies^19,33,34^. Hence, TENS was applied in inert mode with an intensity suitable to induce a slight sensation on the skin, as reported by the subjects, without muscle contraction. In two days, the participant underwent the same TENS application but with different verbal instructions. In particular, in the placebo condition, the experimenter informed the participant that the TENS device was set to active mode, with a stimulation frequency suitable to induce better control of movement speed and accuracy during the task. In the control condition, the experimenter informed the participant that the TENS device was set to an inert mode that served as control and that, despite the slight sensation on the skin, the stimulation frequency could not affect movement execution. To summarize, the same subject was tested in 2 days, one for the placebo condition and one for the control condition, presented with counterbalanced order across participants. Each condition consisted of three sessions (session 1, session 2, and session 3) and, unbeknown to the participants, TENS was applied with the same (inert) frequency. The only difference between conditions was related to the verbal information about the effects of TENS.

### Subjective variables

In addition to the behavioral outcomes (i.e., MT and percentage of error), we decided to monitor subjective variables throughout the experimental procedure. This approach allowed us to investigate any differential effects of the placebo procedure on the behavioral and subjective components of performance. At the end of each session, participants were asked to judge their perception of performance on a 10 cm visual analog scale (VAS), ranging from 0 (“*very bad*”) to 10 (“*excellent*”). This variable allowed us to check whether any behavioral improvement or worsening of performance could be detected by the participants at a subjective level. Moreover, since the task consisted of many repetitions of the reaching movement (i.e., 160 trials), repeated for three consecutive sessions (for a total of 480 trials), we decided to monitor perceived fatigability throughout the procedure. After each session, participants were asked to judge their perception of mental and physical fatigability using a numerical rating scale (NRS), ranging from -3 (“*very tired*”) to 3 (“*very energetic*”), with 0 indicating neutral/indifferent. In addition, to determine whether the TENS application had induced different expectations in the placebo and control conditions, participants were asked to report whether they expected a change in performance before starting the reaching task in sessions 2 and 3, using an NRS ranging from − 3 (“*performance will be much worse than at baseline*”) to + 3 (“*performance will be much better than at baseline*”), with 0 (“*performance will be the same as at baseline*”). Finally, in sessions 2 and 3 soon after having completed the reaching task, participants were asked to evaluate the efficacy of TENS on a VAS from 0 (“*not effective at all*”) to 10 (“*extremely effective*”). The assessment of expectation and perception of TENS efficacy allowed us to test the validity of the placebo procedure in inducing positive expectations and belief in the effect of the treatment.

### Statistical analysis

Before running the statistical analysis, a filter was applied to clean the data of all variables from potential outlier values, defined as values exceeding 3SD the mean value of each block. By applying this filter, outlier values emerged only for MT (0.55% of trials) and were removed from the analysis.

Analyses were performed using SPSS Statistics software (IBM SPSS Statistics 26, SPSS Inc., Chicago, IL). Behavioral data (MT and percentage of error) were normally distributed (Shapiro–Wilk test, *p* > 0.051 for all variables) and were analyzed with parametric tests. Repeated measures analysis of variance (rmANOVA) was conducted with Condition (control and placebo), Session (session 1, session 2, and session 3), and ID (2.5, 3, 3.5, 4, 4.5, 5, 5.5) as within-subject factors. To explore significant effects and interactions, post-hoc comparisons were performed using paired sample t-tests. This statistical plan applied to our experimental design allowed us testing our main hypothesis: shorter MT in the placebo condition compared to the control condition after the application of the placebo device—i.e., in sessions 2 and 3. This main hypothesis could be tested with the interactions Condition × Session and Condition × Session × ID and their respective post-hoc comparisons. We also hypothesized that MT would follow the typical Fitts’ law with longer MT for higher ID (main effect of ID). To test for the impact of target width and target amplitude on performance, we ran additional analyses, as described in the Supplementary Information.

Subjective data (perception of performance, perception of fatigability, expectation scores, and treatment efficacy scores) were analyzed by means of non-parametric tests, due to the ordinal nature of the data. The Friedman test was used to test whether the subjective perception of performance and fatigability varied across sessions (factor session), separately in each condition. Post hoc comparisons were performed using the Wilcoxon signed-rank test. The Wilcoxon signed-rank test was also applied to compare the two conditions (placebo and control), separately in each session and to analyze the expectation scores and treatment efficacy scores, separately in each condition and session. In order to explore if the procedure was effective in inducing positive expectation, expectation scores were also analyzed against 0 by means of the Wilcoxon signed-rank test.

A Bayesian paired sample t-test was used to compare the distribution of different IDs in the two conditions (placebo and control). If the Bayes Factor (BF, here we report BF_10_) is less than 0.33, it represents the absence of a difference in the distribution of the two datasets (confirming the null hypothesis). BF_10_ between 0.33 and 3 is considered inconclusive, i.e., it does not allow for confirmation of the null or the alternative hypothesis. BF_10_ more than 3 supports the alternative hypothesis (significant difference in the distribution of two datasets)^35^.

The effect size of all significant results was calculated with partial eta-square (η_p_^2^) and Hedges’g (as an adjustment to Cohen’s d^36^) for repeated measures ANOVA and paired-sample t-tests, respectively^37^. For nonparametric analysis, the effect size of all significant results was calculated with Kendall’s W and r for Friedman and Wilcoxon signed-rank tests, respectively^38^. The Bonferroni correction for multiple comparisons was applied where necessary. The level of significance was set at *p* < 0.050. Analysis of reliability was performed by Cronbach’s alpha test, by considering the number of measurements of each variable as items.

Behavioral data are reported and represented as mean ± standard error (SE), whereas subjective data are reported as median ± IQR and represented as box plot.

### Ethical approval

All procedures of the experiment followed the institutional ethical standards, in line with the 1964 Declaration of Helsinki and its later amendments or comparable ethical standards. This study was approved by the Ethics Committee of the University of Verona (CARP).

### Consent to participate

Written informed consent was obtained from all individuals who participated in this study.

## Results

### Behavioral data

Analysis of MT revealed a significant effect of Condition (F_(1,23)_ = 6.71, *p* = 0.016, *η*_*p*_^2^ = 0.226), Session (F_(2,46)_ = 11.51, *p* < 0.001, *η*_*p*_^2^ = 0.334), ID (F_(6,138)_ = 396, *p* < 0.001, *η*_*p*_^2^ = 0.945) and, more interestingly, significant interactions Condition × Session (F_(2,44)_ = 40.02, *p* < 0.001, *η*_*p*_^2^ = 0.635) and Condition × Session × ID (F_(12,276)_ = 2.61, *p* = 0.003, *η*_*p*_^2^ = 0.102). Since the interactions encompassed the main factors and provide a new finding to test our main hypothesis, they were further analyzed. Post-hoc comparisons for the Condition × Session interaction revealed that MT was shorter in the placebo than in the control condition in sessions 2 and 3 (for both comparisons, *p* < 0.001, Hedges’ g > 0.64), suggesting a positive influence of the placebo procedure on performance, in line with our main hypothesis. Furthermore, specifically in the placebo condition, MT was shorter in sessions 2 and 3 compared to session 1 and in session 3 compared to session 2 (for all comparisons, *p* < 0.001, g > 0.35). This finding indicates an improvement of performance after the application of the placebo device, when positive expectations were induced.

**Figure 3:**
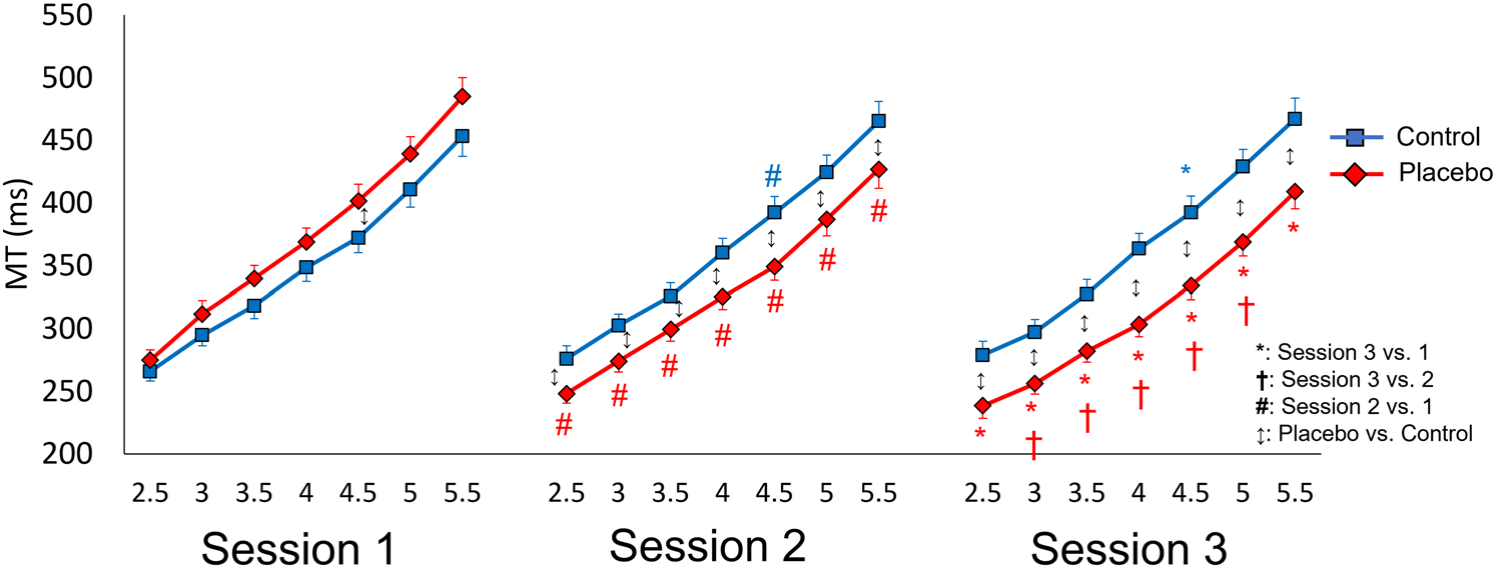
Mean movement time (MT) against different indexes of difficulty in the three sessions for the control (blue line) and placebo (red line) conditions. The indexes of difficulty are reported on the x-axis from 2.5 to 5.5. Asterisks represent significant comparisons between sessions 3 and 1; crosses represent significant comparisons between sessions 3 and 2; hashtags represent significant comparisons between sessions 2 and 1. Up-down arrows represent significant comparisons between the placebo and control conditions. Error bars represent standard errors. The level of significance was set at *p* < 0.05.

Post-hoc comparisons for the Condition × Session × ID interaction confirmed this finding, by revealing that MT was shorter in the placebo than in the control condition in sessions 2 and 3 for all IDs (for all comparisons, *p* < 0.017, g > 0.74), whereas MT was longer in the placebo than in the control condition specifically for ID 4.5 in session 1 (*p* = 0.027, g = 0.68). Moreover, specifically in the placebo condition, MT was shorter in sessions 2 and 3 compared to session 1 for all IDs, and it was shorter in session 3 compared to session 2 for IDs 3 to 5 (for all comparisons, *p* < 0.025, g > 0.83) (Fig. 3). Again, this finding indicates an improvement of performance after the application of the placebo device that persisted even in session 3, specifically for difficult IDs. Conversely, in the control condition, MT was longer in sessions 2 and 3 compared to session 1, only for ID 4.5 (for both comparisons, *p* < 0.047, g > 0.75). Finally, according to Fitts’ law, MT was progressively longer with increasing IDs in both placebo and control conditions and in all sessions (for all comparisons, *p* < 0.016, g > 1.18). This finding indicates that higher IDs were associated with longer MT, while lower IDs were associated with a shorter MT, as typically observed in Fitts’ law tasks.

A Bayesian analysis was performed in each session to compare the distribution of MT in the placebo and control conditions for different IDs. In session 1, the Bayesian analysis supported the null hypothesis for the same IDs or almost similar IDs in the two conditions: ID 2.5 placebo and ID 2.5 control (BF_10_ = 0.254), ID 3 placebo and ID 3.5 control (BF_10_ = 0.178), ID 3.5 placebo and ID 4 control (BF_10_ = 0.217), ID 4 placebo and ID 4.5 control (BF_10_ = 0.164), ID 4.5 placebo and ID 5 control (BF_10_ = 0.2), and finally, ID 5 placebo and ID 5.5 control (BF_10_ = 0.223). The analysis yielded inconclusive results or confirmed the alternative hypothesis for the other paired comparisons (for all, BF_10_ > 0.453). In session 2, Bayesian analysis supported the null hypothesis when comparing higher IDs in the placebo condition with lower IDs in the control condition (with a difference of 0.5 ID), as listed here: ID 3 placebo and ID 2.5 control (BF_10_ = 0.159), ID 3.5 placebo and ID 3 control (BF_10_ = 0.170), ID 4 placebo and ID 3.5 control (BF_10_ = 0.157), ID 4.5 placebo ID and ID 4 control (BF_10_ = 0.326), ID 5 placebo and ID 4.5 control ID (BF_10_ = 0.179), ID 5.5 placebo and ID 5 control (BF_10_ = 0.159). The analysis yielded inconclusive results or confirmed the alternative hypothesis for other paired comparisons (for all, BF_10_ > 2.22). In session 3, the Bayesian supported the null hypothesis when comparing even higher IDs in the placebo condition with lower IDs in the control condition (with a difference of 1 ID), as listed here: ID 3.5 placebo and ID 2.5 control (BF_10_ = 0.166), ID 4 placebo and ID 3 control (BF_10_ = 0.211), ID 4.5 placebo and ID 3.5 control (BF_10_ = 0.217), ID 5 placebo and ID 4 control (BF_10_ = 0.182). The analysis yielded inconclusive results or confirmed the alternative hypothesis for other paired comparisons (for all, BF_10_ > 0.389).

**Figure 4:**
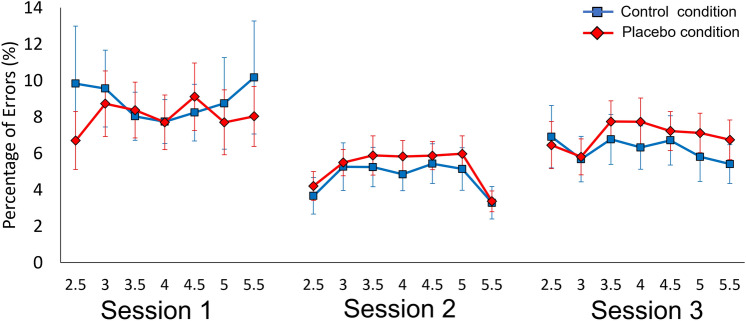
Percentage of error. The mean value of percentage of error against different indexes of difficulty in the three sessions for the control (blue line) and placebo (red line) conditions. The indexes of difficulty are reported on the x-axis from 2.5 to 5.5. Error bars represent standard errors.

Analysis of percentage of error revealed a significant effect of Session (F_(2,46)_ = 5.03, *p* = 0.023, *η*_*p*_^2^ = 0.180), due to a reduction in errors in session 2 with respect to session 1 (*p* = 0.014, g = 0.71) (Fig. 4). Condition, ID, and the interactions were not statistically significant (for all, p > 0.087).

### Subjective data

**Figure 5:**
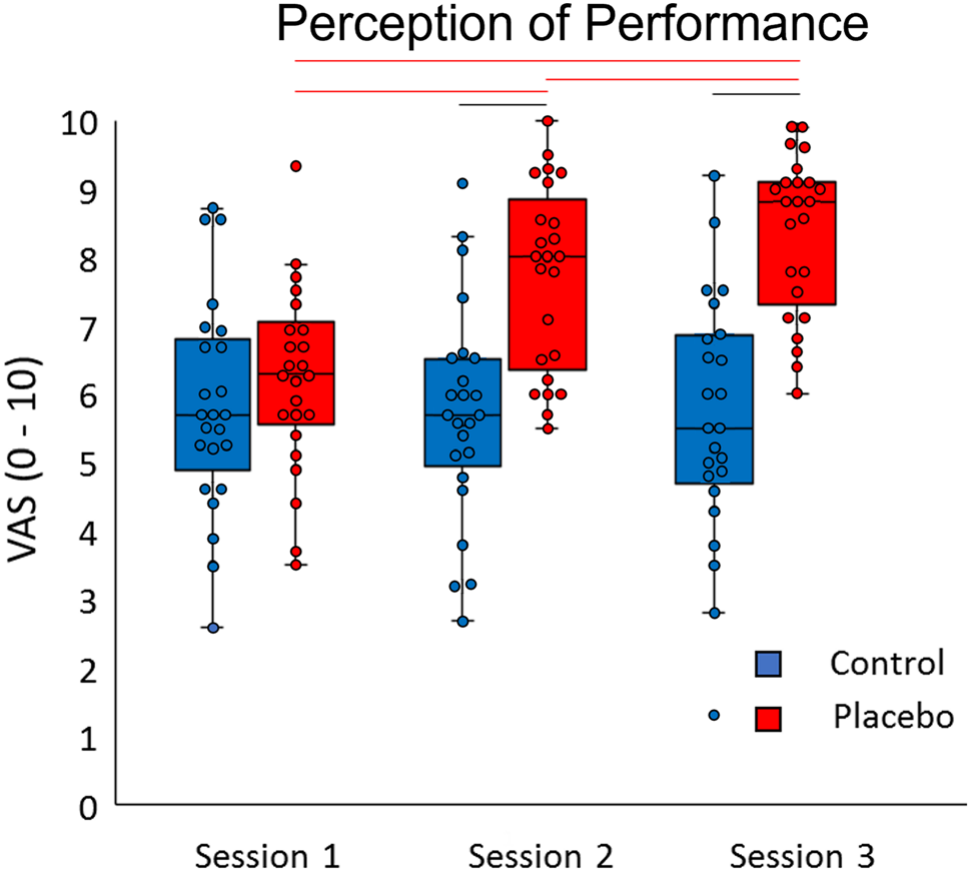
Box plots of the perception of performance on the visual analog scale (VAS). The scores are represented in three sessions for the control (blue box) and placebo (red box) conditions. Red lines represent significant comparisons between sessions in the placebo condition. Black lines show significant comparisons between the control and placebo conditions. Circles represent the subjects’ data. The level of significance was set at *p* < 0.05.

The Friedman test on perception of performance was significant in the placebo condition (χ^2^ = 33.20, df = 2, *p* < 0.001, Kendall’s W = 0.722), but not in the control condition (p = 0.471). Post-hoc comparisons showed that in the placebo condition, the scores were higher in sessions 2 and 3 compared to session 1 and in session 3 compared to session 2 (for all comparisons, *p* < 0.001, r > 0.59) (Fig. 5). The Wilcoxon signed-rank test revealed that scores were higher in the placebo condition compared to the control condition in sessions 2 and 3 (for both comparisons, *p* < 0.001, r > 0.59).

**Figure 6:**
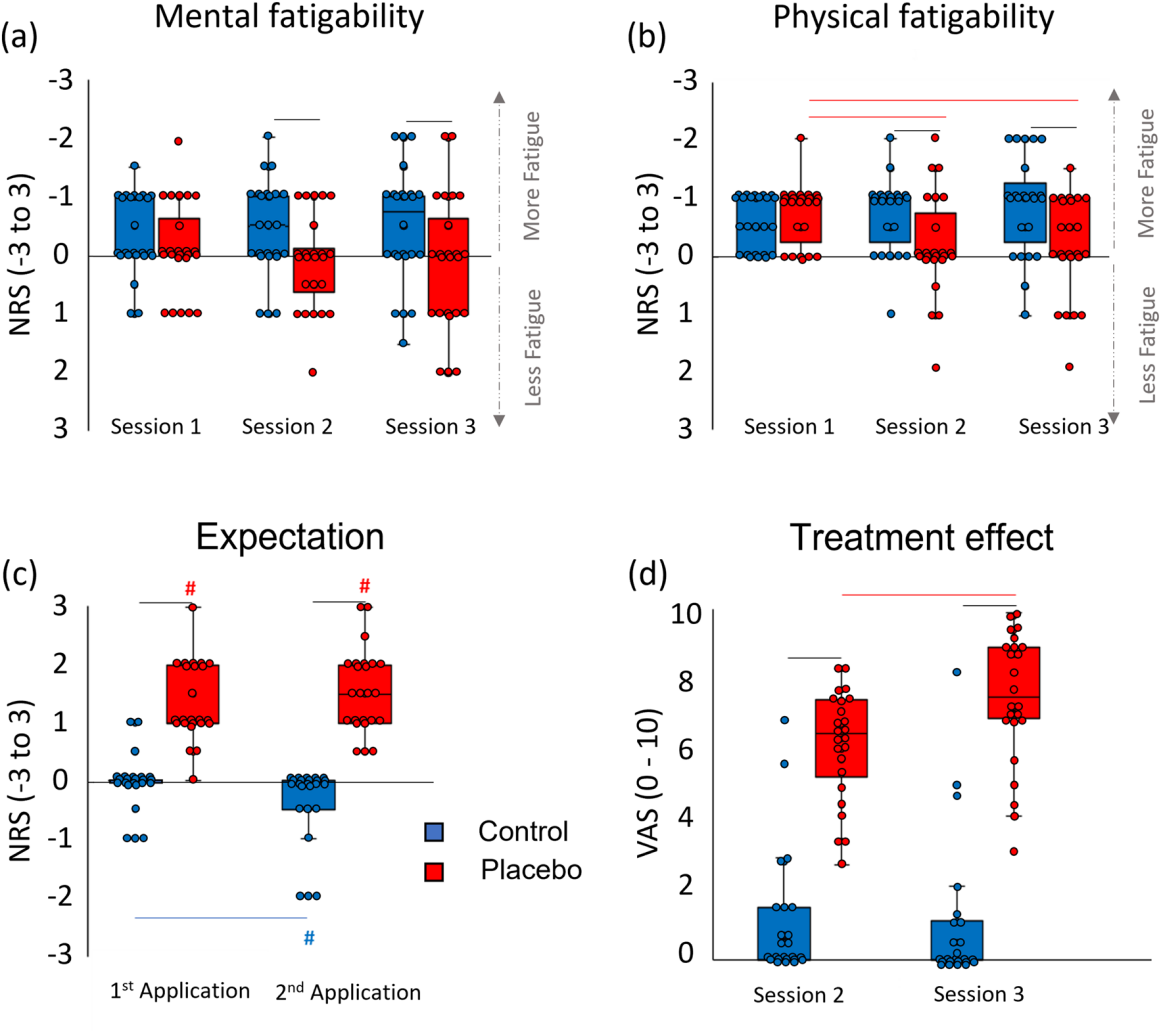
Box plots of the perception of mental and physical fatigability, expectation scores, and treatment effect. The scores of mental (**a**) and physical (**b**) fatigability on the numerical rating scale (NRS) are represented in three sessions for the control (blue boxes) and placebo (red boxes) conditions. The expectation scores (**c**) on the numerical rating scale (NRS) are represented after the first and the second TENS application. The scores of treatment efficacy (**d**) on the visual analog scale (VAS) are represented after sessions 2 and 3. Red lines represent significant comparisons between sessions in the placebo condition. Black lines show significant comparisons between the control and placebo conditions. The blue line shows a significant comparison between sessions in the control condition. The hashtag represents a significant difference from zero. Circles represent the subjects’ data. The level of significance was set at *p* < 0.05.

Analysis of fatigability revealed that participants perceived less mental and physical fatigability in the placebo condition compared to the control condition in sessions 2 and 3 (Wilcoxon, for all comparisons: *p* < 0.020, r > 0.34) (Fig. 6a,b). Moreover, the Friedman test on physical fatigability was significant in the placebo condition (χ^2^ = 6.74, df = 2, p = 0.034, W = 0.147), but not in the control condition (p = 0.159). Post-hoc comparisons showed that in the placebo condition, participants perceived less physical fatigability in sessions 3 and 2 compared to session 1 (for both pairwise comparisons, *p* < 0.048, r > 0.35) (Fig. 6b).

Expectation scores were higher in the placebo compared to the control condition, both for the first and the second TENS application (Wilcoxon, *p* < 0.001, r > 0.61) (Fig. 6c). In the control condition, expectation scores were lower in the second compared to the first TENS application (Z = − 2.64, *p* = 0.010, r = 0.38). Analysis against zero revealed that in the placebo condition, scores were significantly above zero for both TENS applications (*p* < 0.001, r > 0.62). In the control condition, expectation scores were below zero after the second TENS application (*p* = 0.016, r = 0.35).

The perception of treatment efficacy was higher in the placebo than in the control condition for both the first and second TENS applications (for both comparisons, *p* < 0.001, r > 0.62) (Fig. 6d). Furthermore, in the placebo condition, treatment efficacy scores were higher in the second compared to the first TENS application (*p* < 0.001, r = 0.59).

### Reliability scores

The value of Cronbach’s α for MT (42 items) was α = 0.986, and for percentage of error (42 items) was α = 0.946. The value of Cronbach’s α for perception of performance (6 items) was α = 0.890, for mental fatigability (6 items) was α = 0.783, for physical fatigability (6 items) was α = 0.798, for expectation scores (4 items) was α = 0.756, and for perception of treatment efficacy (4 items) was α = 0.794.

## Discussion

This study was aimed at investigating the placebo effect on goal-directed movements performed with the upper limb. According to Fitts’ law, our findings clearly show that movement time was progressively longer with increasing index of difficulty, in both placebo and control conditions. Interestingly, after the application of the placebo treatment in the second and third sessions of the placebo condition, movement time was significantly reduced for all indexes of difficulty compared to the control condition, thus indicating an improvement of performance due to the placebo effect. Moreover, we could observe that the reduction of movement time was even greater after the second than the first placebo intervention. With this regard, after the first placebo application (session 2), the mean movement time for ID 3.5 in the control condition was similar to the mean movement time for ID 4 in the placebo condition. In other words, in the placebo condition we observed a gain in movement time of 0.5 ID, as evidenced by the Bayesian analysis. Interestingly, after the second placebo intervention (session 3), the mean movement time for ID 3.5 in the control condition was similar to the mean movement time for ID 4.5 in the placebo condition. In this case, we observed a gain in movement time of 1 ID in the placebo condition compared to the control condition. Hence, not only movement time was significantly reduced across sessions in the placebo condition, but the reduction of movement time was even doubled after the second application of the placebo treatment. This finding could be indicative of a dose-dependent placebo response, in line with previous studies on different tasks^39^. Our findings also show a clear decrease in the number of errors in the second session in both conditions, likely due to practice.

The subjective data confirmed the efficacy of the placebo procedure in inducing a placebo effect. First, we found that in the placebo condition, participants had positive expectations about performance improvement and judged the treatment as effective. This finding certifies the validity of our procedure in inducing a positive attitude toward the treatment. Second, participants subjectively perceived an improvement in performance across sessions in the placebo condition, while their perception of performance remained stable in the control condition. Third, perception of mental and physical fatigability was lower in the placebo compared to the control condition, indicating that the placebo treatment induced a reduction of fatigability. This is a particularly interesting finding, as it demonstrates that the execution of repeated arm movements did not induce fatigability in the placebo condition. Conversely, participants felt less fatigued in the placebo condition, suggesting that the placebo procedure could have boosted both the behavioral and subjective components of performance.

One could argue that practice could have influenced our findings. If this were the case, we should have observed practice-induced performance improvement across sessions in both conditions (placebo and control). However, no improvement of performance was observed in the control condition, suggesting that practice per se cannot account for our results. Moreover, we can also rule out that the 10 Hz electrical stimulation of TENS per se could have influenced our results for two main reasons. First, performance in the control condition did not change after the two TENS applications, suggesting that TENS per se did not improve performance. This is in line with previous studies in which we applied 10 Hz TENS to control groups with the same modality used in the current study and we did not observe improvement of performance nor changes of neurophysiological parameters^19,33,34^. Second, according to our within-subject design, the same subject was tested in 2 days with the same TENS application (i.e., same frequency of stimulation, same location). Hence, if the electrical stimulation of TENS per se had affected performance or altered sensorimotor mechanisms, this should have occurred in both conditions, being TENS applied in the same way.

A possible explanation for our results could be found in the interplay between feedback and feedforward processes of motor control. Feedback processes are inherently slow, as feedback control uses sensory information to modify ongoing movements^40^. Conversely, feedforward movements can be performed rapidly, as they occur without the online use of sensory feedback and require an internal model of the movement that allow to estimate the future sensory state of the moving limb^41^. Optimal movement control requires a combination of both feedforward and feedback processes^12^. In Fitts’ law tasks, movement control ranges from feedback to feedforward processes, depending on the index of difficulty^40^. For instance, when the task is difficult, that is at higher indexes of difficulty, slow feedback processes prevail, while when the task is easy, that is at lower indexes of difficulty, rapid feedforward processes can occur^40^. We could hypothesize that the positive expectation of being able to perform the task faster because of the treatment, could have changed the participants’ mind-set with regards to task difficulty. In other words, positive verbal suggestion in the placebo condition could have induced participants to shift from feedback to feedforward control even at higher indexes of difficulty, thus shortening the movement time. This speculation should be proven in future studies by adding, for instance, kinematic and neuroimaging recordings during the performance of the reaching movement.

Attention is another potential factor to be considered in explaining our results. Namely, positive expectations induced through the placebo procedure could have acted by modulating the allocation of attentional resources during the task. Conversely, in the control condition, subjects may have had their attention unchanged, due to the lack of expectation. It should be noted that in both conditions (placebo and control), the same treatment was applied to the same body region (i.e., the arm). Hence, what could have eventually modulated the different allocation of attentional resources in the two conditions was not the treatment itself, but rather the different verbal instructions. For instance, it is likely that in the placebo condition, the verbal suggestion about the positive effects of the treatment could have induced participants to expect a specific effect during movement execution, thus orienting their attention toward the body. Interestingly, when attention is directed to the body, the placebo effect appears to be stronger^34,42^. Hence, we could speculate that the placebo effect could have induced shorter movement time, by reallocating attention to the body district relevant for the task. This notion well fits with the evidence that younger adults preferentially use a hand-centered frame of reference when performing a reaching task, like the one adopted in our study^43,44^. These observations notwithstanding, we cannot completely exclude the possibility that the placebo procedure induced attentional allocation toward the goal of the action (i.e., the target) instead of the hand. Previous studies have shown that verbal instructions used to direct the focus of attention can modulate the execution of goal-directed movements. More precisely, an external focus of attention induces a benefit in Fitts’ law tasks compared to an internal focus of attention^45–47^. Whatever the direction of the attentional focus was, we could more generally speculate that the procedure could have acted on the connection between attention and motor action^48^. With regard to this, we know that attention distribution is modulated by the action to be performed^49^. Moreover, when attention is captured by a stimulus, an action plan is also automatically activated to interact with that stimulus^50^. The idea that attention is distributed within an action-centered representation during reaching tasks was first introduced by Tipper and colleagues^44^ and then further confirmed by other authors^51–53^. This theoretical framework leads us to speculate that the placebo procedure could have improved performance by leveraging attentional mechanisms within the action-centered representation. The precise direction of the allocation of attentional resources in our task should be better clarified in future studies, for instance by systematically manipulating the focus of attention (toward the target or toward the body).

The neural mechanisms underlying these effects have not been investigated in our study. However, it is reasonable to speculate that the placebo-induced improvement we observed could be related to the involvement of the brain network involved in the cognitive control of movements. In particular, the cognitive control of motor behaviour requires a complex brain network in which the dorsolateral prefrontal cortex, together with other frontal regions, establishes connections with cortical and subcortical structures involved in motor control, like the primary motor cortex, supplementary motor area, premotor areas, and basal ganglia^54–57^. Interestingly, the dorsolateral prefrontal cortex is involved in elaborating expectation, which is one of the key cognitive mechanisms at the basis of the placebo effect induced by verbal suggestion^58,59^ and has been found to be involved in the placebo-induced increase of force^27^. Hence, by taking all this evidence together, we speculate that the positive expectations induced by the placebo procedure could have enhanced the activity of the dorsolateral prefrontal cortex and favoured its connections with other motor brain regions, thus resulting in the better performance of goal-directed movements observed in the placebo condition.

Fitts’ law has been applied to behavioral data in a variety of motor tasks performed with the upper limb^1,60–63^. A previous study showed that fatigue induces an increase in movement time by maintaining constant the shape of Fitts’ law^9^. In other words, movements were slower to guarantee task success in the presence of fatigue^9^. This finding confirms that behavior is flexible and can adapt quite rapidly to different conditions. With our study, we shed new light on the factors modulating reaching movements by demonstrating that a top-down function, like expectation of benefit induced with verbal suggestion, shortens movement time.

Altogether, these findings indicate that a placebo procedure can positively influence the speed-accuracy trade-off in upper limb reaching movement and expand our understanding of the powerful effects of placebos in the motor domain. Understanding whether a placebo procedure improves goal-directed movements may have important implications in the clinical setting. For instance, the protocol of this study, adapted and modified according to the context and the individual needs, could inspire future applications to boost the recovery of upper-limb movements in patients with motor deficits. During the rehabilitation phase, a patient’s belief of poor performance, the lack of motivation, and the low expectation of improvement might undermine compliance, thus hindering the progress of a full or partial recovery of motor functions. Enhancing patient’s expectation of good motor performance using cognitive strategies might capitalize on the patent’s residual potential, strengthen adherence to the rehabilitation protocols, and lead to better recovery of motor abilities. Hence, we believe that harnessing the placebo effect could eventually inspire future rehabilitation strategies in the long term.

## Supplementary Information

Supplementary Information.

## Data Availability

Data collected for this study are available by request through the corresponding author.
