# Supplementary material for: The placebo effect shortens movement time in goal-directed movements
Source: Sci Rep. 2022 Nov 15;12:19567. doi: 10.1038/s41598-022-23489-y (PMC9666443; doi:10.1038/s41598-022-23489-y)
Supplement: Supplementary file 1 — Supplementary Information. [file 41598_2022_23489_MOESM1_ESM.docx]

**Supplementary information**

**The placebo effect shortens movement time in goal-directed movements**

Mirta Fiorio^1*^, Bernardo Villa-Sánchez^1,2^, Filippo Rossignati^1^, Mehran Emadi Andani^1*^

^1^ Department of Neurosciences, Biomedicine and Movement Sciences, University of Verona, Verona, 37131, Italy

^2^ Center for Mind/Brain Sciences (CIMeC), University of Trento, Rovereto, 38068, Italy

* Prof. Mirta Fiorio, PhD, Tel. +390458425133; email: [mirta.fiorio@univr.it](mailto:mirta.fiorio@univr.it)

* Dr. Mehran Emadi Andani, PhD, Tel. +390458425133; email: [mehran.emadiandani@univr.it](mailto:mehran.emadiandani@univr.it)

**Supplementary analysis and results**

To test for differential impacts of target width and target amplitude on performance in the placebo and control conditions, we derived the MT/ID slope, separately for target width and amplitude [1]. Since we had 4 target widths and 4 target amplitudes, we computed the MT/ID slope in two ways. Precisely, to test for the impact of target width the MT/ID slope (Slope_width_) was derived by keeping constant the target amplitude (e.g., A1=134.4 mm) and by varying the four target widths (16.8, 23.8, 33.6, and 47.5 mm). This slope (Slope_width_A1_) corresponded to a specific IDs range (e.g., in this case, IDs of 4, 3.5, 3, and 2.5 bits, Fig. S1A). The same approach was used by keeping constant the other target amplitudes (i.e., A2 = 190, A3 = 268.7, and A4 = 380 mm) and, for each amplitude, by varying the four target widths (16.8, 23.8, 33.6, and 47.5 mm). These slopes (Slope_width_A2_, Slope_width_A3_, Slope_width_A4_) corresponded to different IDs ranges, one for each target amplitude considered (Fig. S1A). As such, we had four Slope_width_, one for each constant target amplitude.

Similarly, to investigate the effect of target amplitude, the MT/ID slope (Slope_amplitude_) was derived by keeping constant the target width (e.g., W1=47.5 mm) and by varying the four target amplitudes (134.4, 190, 268.7, and 380 mm). This slope (Slope_amplitude_W1_) corresponded to a specific IDs range (e.g., in this case, IDs of 2.5, 3, 3.5, and 4 bits, Fig. S1B). Even in this case, the same approach was used also by keeping constant the other target widths (i.e., W2 = 16.8, W3 = 23.8, and W4 = 33.6 mm) and, for each width, by varying the four target amplitudes (134.4, 190, 268.7, and 380 mm). These slopes (Slope_amplitude_W2_, Slope_amplitude_W3_, Slope_amplitude_W4_) corresponded to different IDs ranges, one for each target amplitude considered (Fig. S1B). As such, we had four Slope_amplitude_, one for each constant target width.

Slope_width_ and Slope_amplitude_ were computed for all participants in each session and condition. Slope_width_ was analysed by means of rmANOVA with Session (session 1, 2, and 3), Condition (placebo, control) and Amplitude (Slope_width_A1_, Slope_width_A2_, Slope_width_A3_, and Slope_width_A4_) as within-subject factors. Slope_amplitude_ was analysed by means of rmANOVA with Session (session 1, 2, and 3), Condition (placebo, control) and Width (Slope_amplitude_W1_, Slope_amplitude_W2_, Slope_amplitude_W3_, and Slope_amplitude_W4_) as within-subject factors.

The effect size of all significant results was calculated with partial eta-square (η_p_^2^) and Hedges’g (as an adjustment to Cohen’s d, [2]) for repeated measures ANOVA and paired-sample t-tests, respectively [3]. The Bonferroni correction for multiple comparisons was applied where necessary. The level of significance was set at p < 0.050. Data are reported and represented as mean ± standard error (SE).

Analysis of Slope_width_ revealed a significant effect of the factor Amplitude (F(3,69) = 9.55, p < 0.001, η_p_^2^= 0.293), because of steeper Slope_width_A4_ (A = 380 mm) compared to Slope_width_A1_ (A = 134.4 mm) and Slope_width_A2_ (A = 190 mm) (for both comparisons, p < 0.002, g > 3.8), indicating steeper slope for higher ID range (more difficult task) than for lower ID ranges (less difficult task). The interaction Session × Condition was also significant (F(2,46) = 9.55, p < 0.001, η_p_^2^= 0.293). Post-hoc comparisons revealed that overall Slope_width_ was steeper in session 1 compared to session 3, specifically in the placebo condition (p = 0.040, g = 2.97) (Fig. S2A). Moreover, in session 3, Slope_width_ was steeper in the control condition compared to the placebo condition (p = 0.004, g = 3.53). Shallower MT/ID slope in session 3 in the placebo condition indicates less increase of MT with increasing task difficulty compared to session 1 and to the control condition.

Analysis of Slope_amplitude_ revealed a significant effects of Session (F(2,46) = 9.97, p < 0.001, *η_p_^2^*= 0.302), due to shallower slope in session 3 compared to sessions 1 and 2 (for both comparisons, p < 0.019, g > 2.1). The factor Width was also significant (F(3,69) = 5.89, p = 0.001, *η_p_^2^*= 0.204), due to steeper Slope_amplitude_W4_ (W = 16.8 mm) compared to Slope_amplitude_W1_ (W = 47.5 mm) and Slope_amplitude_W2_ (W = 33.6 mm) (for both comparisons, p < 0.037, g > 2.06), indicating steeper slope for higher ID range (more difficult task) than for lower ID ranges (less difficult task). The interaction Session × Condition was also significant (F(2,46) = 3.21, p = 0.049, *η_p_^2^*= 0.123). Post-hoc comparisons showed that, specifically in the placebo condition, Slope_amplitude_ was steeper in session 1 compared to session 2 and 3 (for both comparisons, p < 0.008, g = 2.93) (Fig. S2B). Moreover, a tendency was found in session 3, in which Slope_amplitude_ was steeper in the control condition compared to the placebo condition (p = 0.054, g = 1.73). Shallower MT/ID slope in session 2 and 3 in the placebo condition indicates less increase of MT with increasing task difficulty compared to session 1 and to the control condition.


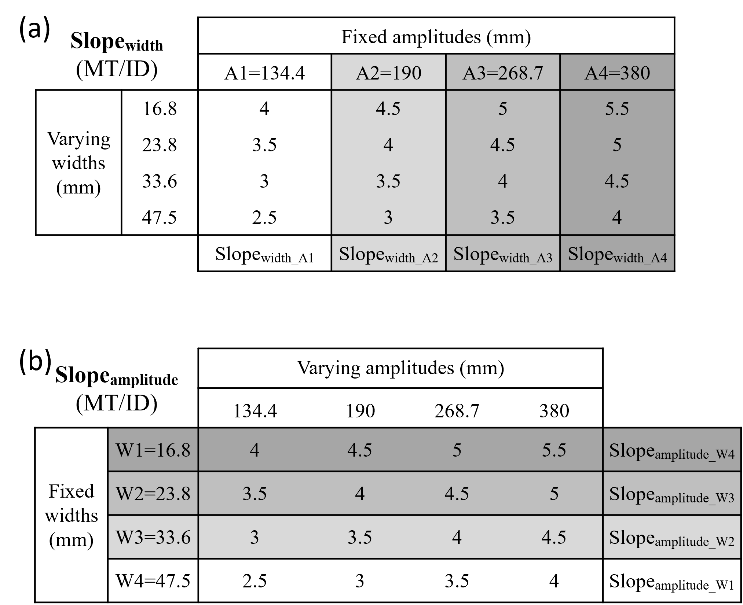


**Fig. S1**. Schematic representation of the computation of slopes (MT/ID). a) Slope_width_ was derived for each target amplitude (A1, A2, A3, A4), by keeping constant the amplitude and varying the width. In this way four Slope_width_ were obtained, one for each target amplitude corresponding to one ID range. b) Slope_amplitude_ was derived for each target width (W1, W2, W3, W4), by keeping constant the width and varying the amplitude. In this way four Slope_amplitude_ were obtained, one for each target width corresponding to one ID range. Figure created by M.F. with Microsoft Power Point, version 2016, URL: <https://www.microsoft.com>).


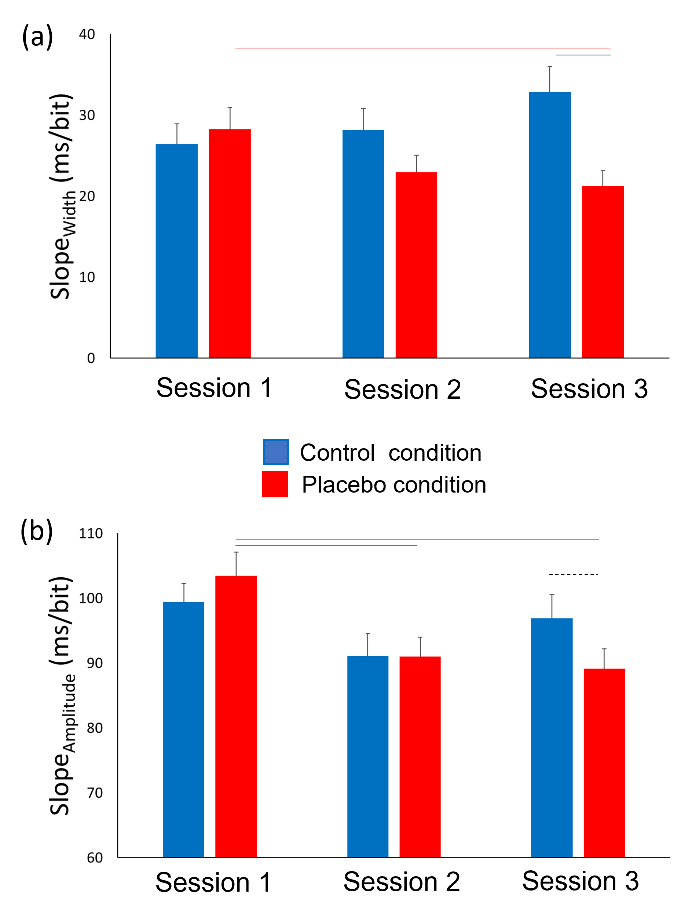


**Fig. S2**. Mean values of the slopes for the significant interaction Session × Condition. a) Slope_width_ was steeper in session 1 compared to session 3, specifically in the placebo condition (red columns), and in the control condition (blue columns) compared to the placebo condition, specifically in session 3. b) Slope_amplitude_ was steeper in session 1 compared to session 2 and 3 in the placebo condition (red columns) and, with a tendency, in the control condition (blue columns) compared to the placebo condition in session 3. Solid lines show statistically significant differences and dashed line shows nearly significant difference (p = 0.054). The level of significance set at *p* < 0.050.

**References**

1. Heath, M., Weiler, J., Marriott, K. A., & Elliott D. Revisiting Fitts and Peterson (1964): Width and Amplitude Manipulations to the Reaching Environment Elicit Dissociable Movement Times. *Canadian Journal of Experimental Psychology*, **65**(4), 259–268. <https://doi.org/10.1037/a0023618> (2011).
2. Lakens, D. Calculating and reporting effect sizes to facilitate cumulative science: a practical primer for t-tests and ANOVAs. *Frontiers in Psychology*, **4**. <https://doi.org/10.3389/fpsyg.2013.00863> (2013).
3. Fritz, C. O., Morris, P. E., & Richler, J. J. Effect size estimates: Current use, calculations, and interpretation. *Journal of Experimental Psychology: General*, **141**(1), 2–18. <https://doi.org/10.1037/a0024338> (2012).
